# Neonatal Antibiotic Treatment Can Affect Stool Pattern and Oral Tolerance in Preterm Infants

**DOI:** 10.3390/life12071043

**Published:** 2022-07-13

**Authors:** Diana Verónica Reyes-García, Arturo Alejandro Canul-Euan, María Antonieta Rivera-Rueda, Claudia Edith Cruz-Alvarado, Luisa Bertha Bermejo-Martínez, Gabriela Arreola-Ramírez, Guadalupe Cordero-González, Sandra Carrera-Muiños, Juan Daniel Diaz-Valencia, Guadalupe Estrada-Gutiérrez, Claudine Irles, Gabriela Gonzalez-Perez

**Affiliations:** 1 Neonatal Intensive Care Unit, National Institute of Perinatology “Isidro Espinosa de los Reyes”, Mexico City 11000, Mexico; dian.reyesg@gmail.com (D.V.R.-G.); marivera1309@yahoo.com.mx (M.A.R.-R.); draclaucruz18@gmail.com (C.E.C.-A.); guadita69@yahoo.com.mx (G.C.-G.); sandracarreram@hotmail.com (S.C.-M.); 2 Department of Developmental Neurobiology, National Institute of Perinatology “Isidro Espinosa de los Reyes”, Mexico City 11000, Mexico; alejandrox07@gmail.com; 3 Department of Immunobiochemistry, National Institute of Perinatology “Isidro Espinosa de los Reyes”, Mexico City 11000, Mexico; bermejol@yahoo.com.mx; 4 Department of Pediatric Follow-Up, National Institute of Perinatology “Isidro Espinosa de los Reyes”, Mexico City 11000, Mexico; gabrielaarreola@hotmail.com; 5 Department of Physiology and Cellular Development, National Institute of Perinatology “Isidro Espinosa de los Reyes”, Mexico City 11000, Mexico; jdanield@yahoo.com (J.D.D.-V.); claudine.irles@inper.gob.mx (C.I.); 6 Research Direction, National Institute of Perinatology “Isidro Espinosa de los Reyes”, Mexico City 11000, Mexico; gpestrad@gmail.com

**Keywords:** empirical antibiotic, neonatal antibiotic, preterm infant stool pattern, meconium, delayed meconium passage, green stools, yellow stools, preterm infant oral feeding, preterm infant feeding tolerance, enteral feeding volumes

## Abstract

Preterm neonates are at high risk of infectious and inflammatory diseases which require antibiotic treatment. Antibiotics influence neonatal gut microbiome development, and intestinal dysbiosis has been associated with delayed gastrointestinal transit. Neonates who take less time to pass meconium have a better tolerance to enteral feeding. We analyzed the effect of neonatal antibiotic treatment on the stool pattern and oral tolerance in 106 preterm infants < 33 weeks gestational age. Neonates were classified in 3 groups according to neonatal antibiotic (ABT) treatment days: no antibiotics, 3–7 d ABT, and ≥8 d ABT. Preterm infants from the ≥8 d ABT group took longer to pass meconium and to start green and yellow stools, took longer to reach 100 and 150 mL/kg/day, and reached reduced volumes in enteral feeds at day of life 14 and 28 than infants from no ABT and 3–7 d ABT groups. Multiple linear regression models showed that neonatal antibiotic treatment, birth weight, invasive mechanical ventilation, surfactant, enteral feeding start day, neonatal parenteral nutrition, and neonatal fasting days are associated with the stool pattern and oral tolerance in preterm infants.

## 1. Introduction

Neonates have an immature immune system and are at high risk of infectious and inflammatory diseases which require antibiotic treatment such as early-onset sepsis (EOS), late-onset sepsis (LOS), and necrotizing enterocolitis (NEC) [1,2,3,4]. Antibiotics are the most administered medications in Neonatal Intensive Care Units (NICUs) worldwide [5,6]. Early-life antibiotic treatment disrupts the developing immune-gut-brain axis in neonates and has been associated with child growth impairment and numerous immune, metabolic, and neurodevelopmental disorders in infancy [7,8,9,10,11,12,13,14,15,16,17,18,19,20,21,22].

Neonatal first bowel movement is called meconium, and after passing meconium, stool goes from dark green to yellow in color. Useful tools have been created to facilitate the description and differentiation between physiological and pathological stool appearance in infants. The Amsterdam Stool Scale in diapered infants describes the consistency, amount, and color of feces in infants [23], and the Diapered Infant Stool Scale also considers the frequency by which watery and mucousy stool occurs in healthy, exclusively breastfed infants [24].

Even though no difference has been shown between the stool characteristics of term and preterm neonates [23], it is known that in both groups of infants the stool pattern changes with the days of extrauterine life, due to the progressive maturity of the intestine and its increased ability to absorb water [23,25]. The appearance of the first meconium stool is useful in determining the proper functioning of the neonate’s gastrointestinal tract. Although it has not been shown to be a predictor of adequate enteral tolerance, it is known that compared to full-term neonates, it can occur later in preterm neonates due to the immaturity of their intestinal motility. It has been observed that neonates who take less time to pass meconium have a better tolerance to enteral feeding [26,27]. Therefore, the time of the last meconium evacuation could be an indicator of enteral tolerance.

Different therapeutic strategies such as use of osmotic contrast agent, saline enemas, and glycerin suppositories have been studied to accelerate meconium passage to increase intestinal motility and improve tolerance to enteral feeding [28,29,30,31,32]. However, none has been shown to be completely effective.

Antibiotics influence neonatal gut microbiome development and affect the density and diversity of bacterial communities [33,34,35,36,37]. This phenomenon is known as intestinal dysbiosis, and it has been associated with delayed gastrointestinal transit in mice [38,39]. Based on this evidence, we hypothesized that neonatal antibiotic treatment could influence the stool pattern of preterm infants and this in turn would affect the oral tolerance of this population.

The aim of this study was to analyze the effect of neonatal antibiotic treatment on the stool pattern and the oral tolerance in preterm infants less than 33 weeks of gestational age.

## 2. Materials and Methods

### 2.1. Study Population and Study Design

Eligibility criteria for the study were preterm infants with birth gestational age (GA) less than 33 weeks, born from mothers > 18 years between March 2018 to August 2019 at the National Institute of Perinatology “Isidro Espinosa de los Reyes” (INPer). This observational prospective longitudinal study with a cohort design included female and male preterm infants < 33 weeks GA, born by vaginal birth and cesarean section, singletons, twins, and triplets, with or without antibiotic treatment during hospital stay in the Intensive and Intermediate Neonatal Care Units of INPer, with signed informed consent from parents. Exclusion criteria were: neonates with congenital abnormalities, diagnosis of autoimmune and genetic diseases and mortality within the first 14 days of life; born from mothers <18 years or mothers with HIV, syphilis, or drug abuse during pregnancy; neonates transferred to other hospitals or without signed informed consent from parents; non-residents of Mexico City and Metropolitan Area. Elimination criteria were neonates diagnosed with necrotizing enterocolitis (NEC) within the first 14 days of life, intestinal malrotation, septic ileus, meconium plug, and mortality within the neonatal period. For the analysis, neonates were classified in 3 groups, considering the neonatal antibiotic (ABT) treatment days: no antibiotics (no ABT), three to seven days of antibiotics (3–7 d ABT), and eight or more days of antibiotics (≥8 d ABT).

### 2.2. Preterm Infant and Maternal Data

Preterm infant and maternal data were retrieved from electronic medical records. Preterm infant variables analyzed were mode of delivery, GA, sex, parity (singletons, twins, triplets), intrauterine growth restriction (IUGR) according to Intergrowth-21st standards, birth weight, birth weight recovery day, prenatal ATB treatment, total ABT treatment days during the neonatal period, total ABT treatment days during hospital stay, prenatal and postnatal steroid, surfactant, magnesium sulfate, non-invasive ventilation (NIV), invasive mechanical ventilation (IMV), length of hospital stay, neonatal and total parenteral nutrition (PN) days, and neonatal fasting days. The neonatal stool pattern was based on stool’s color and described the day of life (DOL) in which meconium started and finished, green stools started, yellow stools started, and the number of days without bowel movements (BM). Meconium was defined as the first stools of the newborn with greenish-black color; dark green and bright green stools were defined as green stools; bright yellow and mustard yellow stools were defined as yellow stools. Preterm infant diagnoses analyzed were culture-confirmed early-onset sepsis (EOS), culture-confirmed late-onset sepsis (LOS), septic shock, necrotizing enterocolitis (NEC) I and II according to Bell’s criteria, atypical pneumonia, moderate and severe bronchopulmonary dysplasia (BPD), patent ductus arteriosus (PDA), anemia, retinopathy of prematurity (ROP), gastroesophageal reflux disease (GERD), and oral intolerance. Birth GA classification considered extremely preterm (26 to <28 GA), very preterm (28 to <32 GA), and moderate preterm (32 to <33 GA) infants. Birth weight classification considered extremely low birth weight (ELBW, birth weight < 1000 g), very low birth weight (VLBW, birth weight 1000 to <1500 g), and low birth weight (LBW, birth weight 1500 to <2500 g) infants. Maternal variables analyzed were maternal age, chorioamnionitis, premature rupture of membranes (PROM) >18 h, undernutrition, overweight, obesity, preeclampsia, high blood pressure, hypothyroidism, diabetes, renal disease, and autoimmune disease.

### 2.3. Antibiotic Usage

Preterm neonates born from mothers with chorioamnionitis and premature rupture of membranes (PROM) greater than 18 h, or from mothers with positive cervical-vaginal culture for Group B beta-hemolytic Streptococci who did not receive treatment, were considered at risk of early-onset sepsis (EOS). These neonates were sampled for hemoculture and then received empirical ABT treatment with ampicillin and amikacin on the day of birth. Neonates were clinically assessed at 72 h of life and if hemoculture and C-reactive protein were negative, empiric ABT treatment was discontinued. Preterm neonates that presented Systemic Inflammatory Response Syndrome (SIRS) criteria (tachycardia, tachypnea, fever or hypothermia, and leukocytosis, leukopenia or bandemia) started antibiotic treatment. Neonates where SIRS occurred within the first 3 days of life (DOL) were clinically diagnosed with EOS and received 7 days of ampicillin and amikacin; whereas neonates where SIRS occurred after the first 3 DOL were clinically diagnosed with late-onset sepsis (LOS) and received 7 to 10 days of piperacillin, tazobactam and vancomycin. Neonates with septic shock received 7 to 14 days of meropenem, and neonates with atypical pneumonia (due to Mycoplasma, Ureaplasma and Chlamydia) received 14 days of clarithromycin. Neonates with NEC II were treated for 7 days with piperacillin, tazobactam and vancomycin. Within the 3–7 d ABT study group, 72.5% of neonates started ampicillin and amikacin treatment within the first week of life, whereas 21.6% started piperacillin, tazobactam and vancomycin treatment after the first 3 DOL. Within the ≥8 d ABT study group, 65.5% of neonates started ampicillin and amikacin treatment within the first week of life, whereas 34.5% started piperacillin, tazobactam and vancomycin treatment after the first 3 DOL. Only 3 neonates within the study cohort belonging to the 3–7 d ABT study group started clarithromycin treatment in the fourth week of life.

### 2.4. Feeding Practices

INPer is a third level health center and a national perinatology referral center that attends high-risk pregnancies following Institutional and International Neonatology Guidelines. The institution has a human milk bank certified by the Quality Control Laboratory for Human Milk managed by the Brazilian Network of Human Milk Banks. Feeding practices currently used in the institute are: first choice, mother’s own milk (MOM); second choice, pasteurized donor human milk (PDHM), and third choice, preterm infant formula (PF). Stable neonates start trophic feedings within 24 h of birth, whereas neonates with IUGR and altered placental blood flow are kept in a fasting state. Enteral feedings are contraindicated during abdominal distension, abdominal obstruction, and bile gastric residuals in orogastric tubes, whereas one or two feedings are deferred during packed red blood cells transfusion. In neonates < 1500 g increases in milk feeds are 12.5 mL/kg/day, and for neonates > 1500 g, they are 25 mL/kg/day. Fortified human milk (FHM) with a bovine-based milk fortifier at 1:50 concentration is initiated after enteral feeds reach 100 mL/kg/day, and after three days of adequate tolerance, the fortifier concentration in human milk is increased to 1:25. Four neonatal feeding regimens were identified: (1) exclusive human milk (HM, includes MOM and/or PDHM), (2) HM + FHM, (3) HM + FHM + PF, and (4) HM + PF. The enteral feeding start day, DOL to reach 100 and 150 mL/kg/day in enteral feeds, and the volume of enteral feeds at 14 and 28 DOL were retrieved from electronic medical records.

### 2.5. Statistical Analysis

Descriptive statistics are reported as mean ± standard deviation (SD) for continuous variables, or frequencies (%) for categorical variables. All numerical variables were assessed for normality with the Shapiro–Wilk test, and no outliers were removed from the analysis. One-way ANOVA with Sidak’s post hoc test or Kruskal–Wallis test with Dunn’s post hoc test were used to compare three means of numerical normally or non-normally distributed variables, respectively. Fisher’s exact test and Chi-Square test were used to compare the frequencies of categorical variables as appropriate. For simple regression analysis of non-normally distributed continuous variables, Spearman correlation was used. Multiple linear regression models were used to analyze if there was a relationship between neonatal antibiotic treatment and the study outcomes: meconium finish day, green stool start day, yellow stool start day, DOL to reach 100 and 150 mL/kg/day in enteral feeds, and volume of enteral feeds at DOL 14 and 28. Four multiple linear regression models were built up to sequentially add maternal and infant variables that could influence the stool pattern and oral tolerance in preterm infants. The variables included in the first model were sex (female, male), birth GA classification (extremely preterm, very preterm, moderate preterm), birth weight classification (ELBW, VLBW, LBW), parity (singleton, twins, triplets), and neonatal ABT treatment classification (no ABT, 3–7 d ABT, ≥8 d ABT). The second model considered the same variables as model 1 with the addition of IMV, postnatal steroid, and surfactant. The third model considered the same variables as model 2 with the addition of chorioamnionitis, and PROM > 18 h. The fourth model considered the same variables as model 3 with the addition of enteral feeding start day, neonatal fasting days, neonatal PN days, and feeding regimen classification (HM, HM + FHM, HM + FHM + PF, HM + PF). Statistical analysis and graphics were performed using GraphPad Prism (San Diego, CA, USA) version 9.3.1 and SPSS (Chicago, IL, USA) version 28.0.1.1. A *p*-value less than 0.05 was considered statistically significant and denoted with asterisk symbols as follows: *p* < 0.05 (*), *p* < 0.01 (**), *p* < 0.001 (***), and *p* < 0.0001 (****).

## 3. Results

### 3.1. Study Population

**Figure 1:**
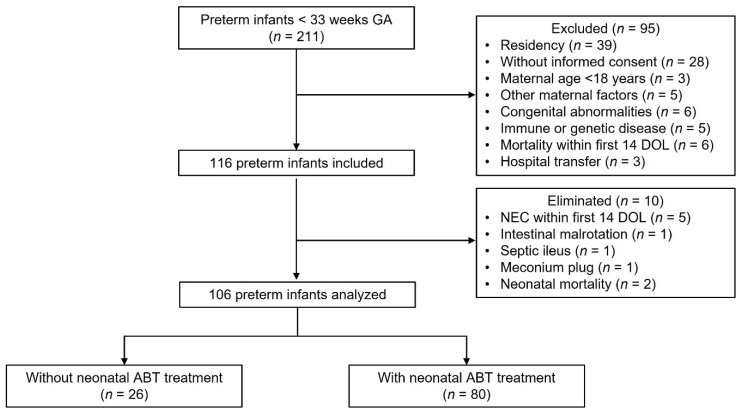
Flow diagram of preterm infants analyzed. Two hundred eleven preterm infants < 33 weeks gestational age were eligible to participate in the study. Ninety-five neonates were excluded, 116 neonates were included, and 10 neonates were eliminated, resulting in 106 neonates analyzed of which 26 were not treated with antibiotics, and 80 were treated with antibiotics during the neonatal period. GA (gestational age), *n* (number), DOL (day of life), ABT (antibiotic).

Between March 2018 and August 2019, 211 preterm infants < 33 weeks GA born from mothers > 18 years were eligible to participate in this observational prospective longitudinal study with a cohort design. Figure 1 shows criteria and number of preterm infants excluded and eliminated from the study. We analyzed 106 preterm infants and classified them in three study groups according to the days they received ABT treatment during the neonatal period: no antibiotics (no ABT, *n* = 26), three to seven days of ABT (3–7 d ABT, *n* = 51), and eight or more days of ABT (≥8 d ABT, *n* = 29).

During the neonatal period, preterm infants from the 3–7 d ABT and the ≥8 d ABT groups received average antibiotic days of 5.5 days and 14.8 days, respectively. Six out of 26 neonates from the no ABT group, 32 out of 51 neonates from the 3–7 d ABT group, and 23 out of 29 neonates from the ≥8 d ABT received additional days of ABT treatment after the neonatal period. During the hospital stay, preterm infants from the no ABT, the 3–7 d ABT and the ≥8 d ABT groups received average antibiotic days of 3.5 days, 15.6 days, and 27.2 days, respectively (Table 1).

### 3.2. Demographic, Nutritional, Maternal, and Clinical Data from Preterm Infants

**Figure 2:**
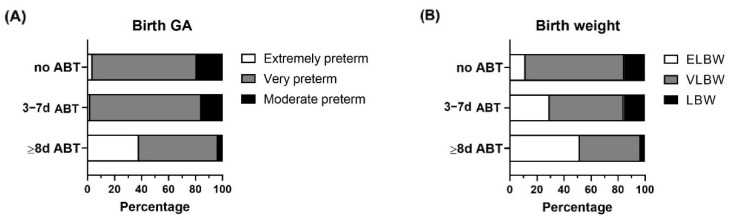
Birth gestational age and birth weight categorization within the study groups. Preterm infants < 33 weeks gestational age (GA) were classified according to neonatal antibiotic treatment days in 3 groups: no antibiotics (no ABT, *n* = 26), three to seven antibiotic days (3–7 d ABT, *n* = 51), and eight or more antibiotic days (≥8 d ABT, *n* = 29). (**A**) Birth GA classification (extremely preterm, very preterm, moderate preterm), (**B**) Birth weight classification, extremely low birth weight (ELBW), very low birth weight (VLBW), low birth weight (LBW).

Preterm infants from the cohort did not have differences in mode of delivery, sex, parity, IUGR, prenatal steroids, and the neuroprotectant magnesium sulfate (Table 1). Neonates that received ≥8 days of neonatal ABT treatment had lower birth gestational age and birth weight and had higher frequencies of extremely preterm and ELBW infants than those who did not receive ABTs or received 3 to 7 days of ABT treatment (Table 1 and Figure 2). All preterm infants recovered their birth weight between 10 and 11 DOL.

In addition, neonates that received ≥8 days of neonatal ABT treatment, received more surfactant, postnatal steroids, IMV, and had longer hospital stay than those who did not receive or received 3 to 7 days of ABT treatment (Table 1).

There were not inter-group differences in feeding practices in preterm infants from the cohort, with all infants starting enteral feeds on average DOL 1.5; 11.5 to 17.2% were fed exclusively HM (MOM and/or PDHM), 42.3 to 58.6% were fed HM and FHM, 15.7 to 26.9% were fed HM and PF, and 6.9 to 29.4% were fed HM, FHM and PF (Table 1). Preterm infants from the ≥8 d ABT group had more neonatal fasting days than those from the no ABT and 3–7 d ABT groups (Table 1). Preterm infants that received neonatal ABT treatment had more neonatal and total parenteral nutrition (PN) days than those who did not received neonatal antibiotics (Table 1).

Mothers in the three groups had an average age of 28 to 30 years and there were no differences in chorioamnionitis, PROM >18 h, pregestational nutritional status (most mothers with healthy weight or overweight), preeclampsia, high blood pressure, hypothyroidism, diabetes, renal disease, or autoimmune disease (Table 1).

There were no differences in the incidence of culture-confirmed EOS, NEC I and II, severe BPD, PDA, anemia, GERD, and oral intolerance between the study groups (Table 2). Preterm infants who received 8 or more days of neonatal antibiotic treatment had higher incidence of culture-confirmed LOS, septic shock, atypical pneumonia, moderate BPD and ROP compared with those that did not or received 3 to 7 days of neonatal ABT treatment (Table 2).

### 3.3. Effect of Neonatal Antibiotic Treatment on Neonatal Stool Pattern and Feeding Tolerance in Preterm Infants

**Figure 3:**
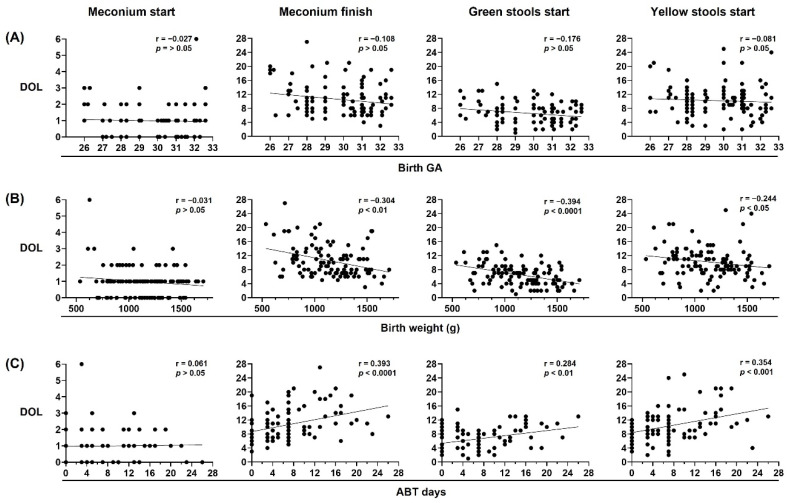
Birth weight and neonatal antibiotic treatment are major contributors to neonatal stool pattern in preterm infants. Neonatal stool pattern from preterm infants < 33 weeks gestational age (GA) at birth (*n* = 106). Day of life (DOL) on which meconium started, meconium finished, green stools started, and yellow stools started were compared with (**A**) birth GA, (**B**) birth weight in grams (g), and (**C**) antibiotic (ABT) days administered during neonatal period. Simple linear regression analysis; Spearman correlation (r) and *p*-values (*p*) are shown.

To evaluate the stool pattern in preterm infants we analyzed the DOL on which meconium started and finished, and green stools and yellow stools started. First, we assessed the influence of birth GA, birth weight and neonatal ABT treatment days on the stool pattern in preterm infants. Our results showed that birth GA was not correlated with the stool pattern, and that birth weight and neonatal ABT treatment days were inversely and directly correlated with the DOL on which meconium finished, and when the green stools and yellow stools started, respectively (Figure 3).

**Figure 4:**
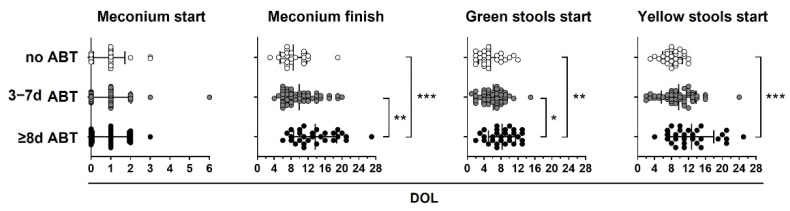
Neonatal antibiotic treatment equal to or greater than 8 days delay in meconium finish, green start, and yellow start stools in preterm infants. Preterm infants < 33 weeks gestational age (GA) were classified according to neonatal antibiotic treatment days in three groups: no antibiotics (no ABT, white circles, *n* = 26), three to seven antibiotic days (3–7 d ABT, gray circles, *n* = 51), and eight or more antibiotic days (≥8 d ABT, black circles *n* = 29). The day of life (DOL) in which meconium started, meconium finished, green stools started, and yellow stools started is shown. Kruskal–Wallis test with Dunn’s post hoc test, *p* < 0.05 (*), *p* < 0.01 (**), *p* < 0.001 (***).

Additionally, we analyzed the stool pattern in the no ABT, 3–7 d ABT and ≥8 d ATB study groups. Preterm infants that did not receive or received 3 to 7 days of neonatal ABT treatment had similar stool patterns. Preterm infants who received 8 or more days of neonatal ABT treatment took a longer time to meconium passage and to start green stools and yellow stools than infants that did not receive or received 3 to 7 days of neonatal ABT treatment (Table 3 and Figure 4).

In Table 3, we describe the average DOL on which preterm infants from the study groups started and finished meconium, started green and yellow stools, and the days without bowel movements (BM) during the neonatal period. On average, all preterm infants started meconium on DOL one, and neonates from the no ABT and 3–7 d ABT groups finished meconium on DOL 8.5 and 9.9, respectively, whereas neonates from the ≥8 d ABT group did it on DOL 13.7 (Table 3). Neonates from the no ABT and 3–7 d ABT groups started green stools on DOL 5.5 and 6.2, respectively, whereas neonates from the ≥8 d ABT group did it on DOL 8.2 (Table 3). Finally, neonates from the no ABT and 3–7 d ABT groups started yellow stools on DOL 8.2 and 9.7, respectively, whereas neonates from the ≥8 d ABT group did it on DOL 12.8 (Table 3). Additionally, neonates from the ≥8 d ABT group had an average of 3 days without bowel movements whereas neonates from the no ABT and 3–7 ABT groups had 1.5 days (Table 3).

**Figure 5:**
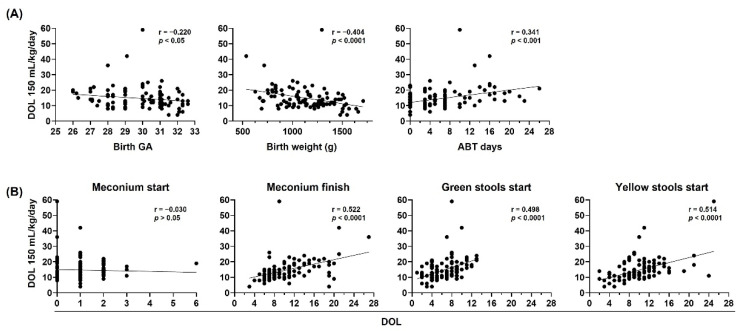
Birth gestational age, birth weight, neonatal antibiotic treatment, and neonatal stool pattern correlate with oral tolerance in preterm infants. The day of life (DOL) to reach 150 mL/kg/day in preterm infants < 33 weeks gestational age (GA) (*n* = 106) was compared with (**A**) birth GA (left), birth weight in grams (g) (center), and antibiotic (ATB) days administered during neonatal period (right), (**B**) DOL on which meconium started, meconium finished, green stools started, and yellow stools started. Simple linear regression analysis, Spearman correlation (r) and *p*-values (*p*) are shown.

Afterwards, we sought to determine the influence of birth GA, birth weight, neonatal antibiotic treatment days, and stool pattern on the DOL in which preterm infants reached 150 mL/kg/day enteral feeds (Figure 5). Our results showed that birth GA and birth weight versus neonatal antibiotic treatment days, inversely and directly correlated with oral tolerance, respectively (Figure 5A). In addition, the DOL on which meconium finished, green stools started, and yellow stools started directly correlated with oral tolerance (Figure 5B).

**Figure 6:**
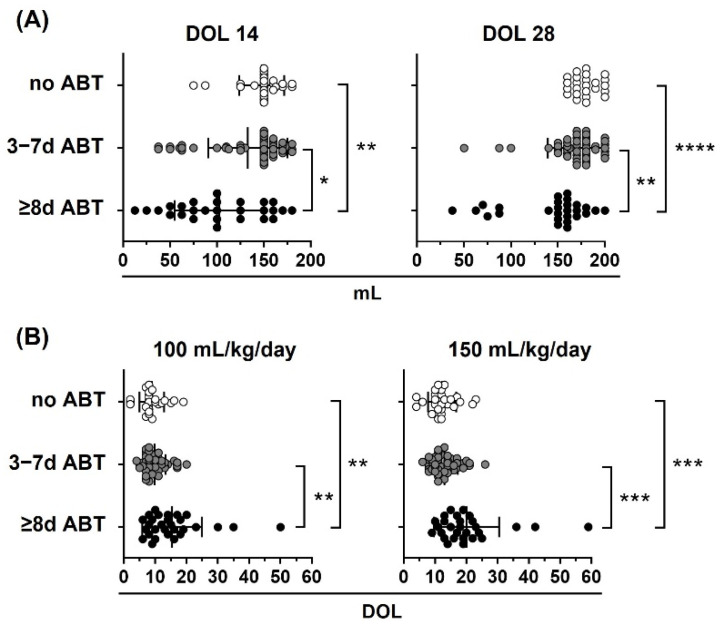
Neonatal antibiotic treatment affects oral tolerance in preterm infants. Preterm infants < 33 weeks gestational age (GA) were classified according to neonatal antibiotic treatment days in three groups: no antibiotics (no ABT, white circles, *n* = 26), three to seven antibiotic days (3–7 d ABT, gray circles, *n* = 51), and eight or more antibiotic days (≥8 d ABT, black circles, *n* = 29). (**A**) Oral volume reached at 14 and 28 days of life (DOL), (**B**) DOL to reach 100 and 150 mL/kg/day. Kruskal–Wallis test with Dunn’s post hoc test; *p* < 0.05 (*), *p* < 0.01 (**), *p* < 0.001 (***), *p* < 0.0001 (****).

Finally, we analyzed the oral tolerance in the no ABT, 3–7 d ABT and ≥8 d ATB study groups (Figure 6). Preterm infants that did not receive neonatal antibiotic treatment and those who were treated with ATB for 3 to 7 days reached similar volumes in oral feeds at DOL 14 and 28 (Figure 6A), and reached 100 and 150 mL/kg/day in enteral feeds in similar DOL (Figure 6B). In contrast, preterm infants who were treated with ABT for 8 or more days, reached reduced volumes in oral feeds at DOL 14 and 28 (Figure 6A), and took longer to reach 100 and 150 mL/kg/day in enteral feeds (Figure 6B) than infants that did not receive ABTs or received 3 to 7 days of ABT treatment.

### 3.4. Multiple Linear Regression Models Show the Relationship of Neonatal Antibiotic Treatment with Meconium Passage, Yellow Stool Start Day, and DOL to Reach 150 mL/kg/day in Preterm Infants

To further analyze if other infant factors could also influence the stool pattern and oral tolerance in preterm infants, we built up four multiple linear regression models to take into consideration the underlying clinical condition of the neonates, and nutritional factors. The study outcomes were meconium finish day, green stools start day, yellow stools start day, DOL to reach 100 and 150 mL/kg/day in enteral feeds, and volume of enteral feeds at DOL 14 and 28. The variables included in the first model were sex (female, male), birth GA classification (extremely preterm, very preterm, moderate preterm), birth weight classification (ELBW, VLBW, LBW), parity (singleton, twins, triplets), and neonatal ABT treatment classification (no ABT, 3–7 d ABT, ≥8 d ABT). The second model considered the same variables as model 1 with the addition of IMV, postnatal steroid, and surfactant. The third model considered the same variables as model 2 with the addition of chorioamnionitis, and PROM >18 h. The fourth model considered the same variables as model 3 with the addition of enteral feeding start day, neonatal fasting days, neonatal PN days, and feeding regimen classification (HM, HM + FHM, HM + FHM + PF, HM + PF).

Birth gestational age, parity, postnatal steroid, chorioamnionitis, and feeding regimen were not associated with the stool pattern and oral tolerance in preterm infants (Table 4, Table 5, Table 6, Table 7, Table 8, Table 9 and Table 10).

Neonatal antibiotic treatment and PROM >18 h had a strong association with the meconium finish day (Table 4).

Both neonatal antibiotic treatment and birth weight lost association with the green stools start day after accounting for nutritional factors among which the enteral feeding start day had a marginal association (Table 5).

Neonatal antibiotic treatment had a strong association with the yellow stools start day (Table 6).

Neonatal antibiotic treatment lost association with DOL to reach 100 mL/kg/day after accounting for nutritional factors among which the neonatal parenteral nutrition and fasting days could have an association (Table 7).

Neonatal antibiotic treatment conserved association with DOL to reach 150 mL/kg/day after accounting for nutritional factors among which the neonatal parenteral nutrition days had an association (Table 8).

Both, neonatal antibiotic treatment and birth weight, lost association with the volume of enteral feeds reached at DOL 14 after accounting for nutritional factors among which the neonatal parenteral nutrition days had a strong association (Table 9).

Finally, multiple factors were associated with the volume of enteral feeds reached at DOL 28 among which the invasive mechanical ventilation, the surfactant, the enteral feeding start day, the neonatal parenteral nutrition and fasting days had a strong association, whereas neonatal antibiotic treatment and sex lost association (Table 10).

## 4. Discussion

Usually, the administration of empirical antibiotic treatment to preterm neonates within the first hours of life is decided based on maternal infection history (chorioamnionitis, PROM, GBS infection) to avoid the risk of EOS. Even though cultured-confirmed EOS is uncommon, empirical antibiotic treatment is continued when neonates exhibit SIRS criteria. We found a low incidence of cultured-confirmed EOS (3.6%), only 2 out of 56 neonates that initiated ampicillin and amikacin treatment during the first 3 DOL had a positive bacterial culture.

Neonatal sepsis is often over diagnosed and making the decision to discontinue empirical antibiotic treatment in preterm infants who are highly vulnerable to infection becomes difficult, and often the regimen continues regardless of the clinical evolution. With few elements to support the diagnosis of bacterial sepsis, antibiotics ideally should be discontinued as quickly as possible, not 48 to 72 h after antibiotic initiation, as it is usually the case. The continuous struggle to find reliable tools to demonstrate a bacterial infection leads pediatricians to maintain unnecessary antibiotic schemes, which can increase the risk of short- and long-term diseases in preterm infants. Therefore, a call is made to improve bacterial diagnostic methods for neonatal sepsis to better adapt antibiotic treatments. By these means, associated risks of using antibiotics might be reduced.

Neonates who take less time to pass meconium have a better tolerance to enteral feeding [26,27], and given that antibiotics affect the density and diversity of bacterial communities [33,34,35,36,37], and intestinal dysbiosis has been associated with delayed gastrointestinal transit in mice [38,39], we hypothesized that neonatal antibiotic treatment could affect the stool pattern and oral tolerance in preterm infants.

Our results showed that preterm infants that received antibiotics for 8 or more days during the neonatal period took longer to pass the meconium and to start green stools and yellow stools. Besides, they took longer to reach 100 and 150 mL/kg/day in enteral feeds and reached reduced volumes in enteral feeds at DOL 14 and 28 than infants that did not receive or received 3 to 7 days of antibiotics.

Bearing in mind that antibiotics delay meconium passage and the transition to green stools and yellow stools in preterm infants, it becomes necessary to focus on the stool pattern in this vulnerable population. This might trigger a reflection based on the impact of antibiotics on intestinal microbiome composition and gastrointestinal motility. With a simple strategy of monitoring stool patterns, it might be possible to predict if the infants will adequately tolerate enteral feeding. By the early detection of stool altered patterns, pediatricians could be able to change treatment strategies or strengthen preventive measures.

Multiple linear regression models identified neonatal antibiotic treatment, PROM > 18 h, birth weight, invasive mechanical ventilation, surfactant, start day of enteral feedings, parenteral nutrition and fasting days as variables associated with the stool pattern, and oral tolerance in preterm infants. Neonatal antibiotic treatment was directly associated with meconium finish day, yellow stools start day, and the DOL to reach 150 mL/kg/day. Although neonatal antibiotic treatment had association with green stools start day, the DOL to reach 100 mL/kg/day, and volume of enteral feeds reached at DOL 14, these associations were lost when nutritional factors were taken in consideration. These findings suggest that neonatal antibiotic treatment could influence on the stool pattern and oral tolerance on preterm infants mainly during the first two weeks of life. The inverse association of PROM >18 h with the meconium finish day could suggest that the fetal distress caused by the PROM could affect the meconium passage in preterm infants. Although birth weight was associated with green stools start day, the DOL to reach 150 mL/kg/day, and the volume in enteral feeds reached at DOL 14, these associations were lost when underlying clinical and nutritional factors were taken into consideration. Given that the preterm infants from the three study groups started enteral feedings on average on day 1.5, it was unexpected to find this variable associated with the volume of enteral feeds reached at DOL 28. However, when analyzing the frequency of neonates from each group starting enteral feeds on the day of birth and on day one, we found higher frequencies of neonates starting enteral feeds on the day of birth and lower frequencies of neonates starting enteral feeds on DOL one within the 3–7 d ABT and ≥8 d ABT groups (15.7% birth day and 43.1% day one, 20.7% birth day and 44.8% day one, respectively) compared with neonates without ABT treatment (7.7% birth day and 61.5% day one). Finally, this study shows that the enteral feeding tolerance at the end of the neonatal period is determined by a complex association of multiple neonatal factors. Invasive mechanical ventilation, neonatal parenteral nutrition and fasting days had an inverse association with the volume of enteral feeds reached at DOL 28, whereas surfactant and the enteral feeding start day has a direct association.

This study identifies neonatal antibiotic treatment as a new factor contributing to the stool pattern and oral tolerance of preterm infants.

## 5. Conclusions

Neonatal antibiotic treatment in preterm infants of less than 33 weeks gestation delays meconium passage and the start of yellow stools, and this altered stool pattern can be associated with impaired enteral feeding. However, here we report the intricate association between multiple neonatal factors such as antibiotic treatment, birth weight, invasive mechanical ventilation, surfactant, start of enteral feedings, neonatal parenteral nutrition and fasting days as critical factors that influence oral tolerance in preterm infants.

## Figures and Tables

**Table 1:** Demographic, nutritional, and maternal data from study cohort.

Neonatal Variable	No ABT (*n* = 26)	3–7 d ABT (*n* = 51)	≥8 d ABT (*n* = 29)	*p*-Value
Demographic				
C-section (%)	24 (92.3)	47 (92.2)	22 (75.9)	0.102
GA (weeks)	30.5 ± 1.6	30 ± 1.6	28.8 ± 1.9	0.0008
Female (%)	13 (50)	20 (39.2)	14 (48.3)	0.589
Singleton (%)	15 (57.7)	37 (72.5)	24 (82.8)	0.118
Twins (%)	6 (23.1)	8 (15.7)	4 (13.8)	0.632
Triplets (%)	5 (19.2)	6 (11.8)	1 (3.4)	0.192
IUGR (%)	6 (23.1)	13 (25.5)	12 (41.4)	0.236
Birth weight (g)	1267 ± 221.5	1150 ± 268.8	998 ± 256	0.0007
Birth weight recovery days	11 ± 3.8	10.3 ± 4.3	10.3 ± 2.9	0.567
Prenatal steroid (%)	18 (69.2)	44 (86.3)	25 (86.2)	0.145
Postnatal steroid (%)	4 (15.4)	14 (27.5)	14 (48.3)	0.025
Surfactant (%)	9 (34.6)	28 (54.9)	22 (75.9)	0.009
Magnesium sulfate (%)	19 (73.1)	36 (70.6)	24 (82.8)	0.477
NIV (%)	26 (100)	50 (98.0)	29 (100)	1.0
IMV (%)	4 (15.4)	29 (56.9)	22 (75.9)	<0.0001
Prenatal ABT (%)	22 (84.6)	45 (88.2)	23 (79.3)	0.566
Neonatal ABT days	0	5.5 ± 1.7	14.8 ± 4.5	-
Total ABT days	3.5 ± 7.4	15.6 ± 9.4	27.2 ± 19.1	<0.0001
Hospital stay days	48.7 ± 19.3	64.3 ± 24.2	73 ± 28.2	0.001
Nutritional				
Enteral feeding start day	1.5 ± 1.2	1.5 ± 1.1	1.5 ± 1.9	0.823
HM (%)	3 (11.5)	6 (11.8)	5 (17.2)	0.755
HM + FHM (%)	11 (42.3)	22 (43.1)	17 (58.6)	0.348
HM + FHM + PF (%)	5 (19.2)	15 (29.4)	2 (6.9)	0.056
HM + PF (%)	7 (26.9)	8 (15.7)	5 (17.2)	0.474
Neonatal PN days	11 ± 7.3	16.1 ± 8.1	19.8 ± 6.5	0.0002
Total PN days	12.5 ± 11.3	18.7 ± 13.4	24.7 ± 16.1	0.0001
Neonatal fast days	1.2 ± 2.1	1.2 ± 1.6	2.8 ± 3.1	0.025
Maternal				
Maternal age (years)	27.8 ± 6.9	28 ± 5.6	30.8 ± 6.6	0.169
Chorioamnionitis (%)	2 (7.7)	6 (11.8)	5 (17.2)	0.553
PROM >18 h (%)	1 (3.8)	12 (23.5)	4 (13.8)	0.087
Healthy weight * (%)	10 (38.5)	21 (41.2)	13 (44.8)	0.905
Underweight * (%)	0	2 (3.9)	1 (3.4)	0.801
Overweight * (%)	13 (50.0)	17 (33.3)	8 (27.6)	0.195
Obese * (%)	3 (11.5)	11 (21.6)	7 (24.1)	0.458
Preeclampsia (%)	13 (50.0)	18 (35.3)	16 (55.2)	0.182
High blood pressure (%)	4 (15.4)	4 (7.8)	7 (24.1)	0.138
Hypothyroidism (%)	5 (19.2)	6 (11.8)	3 (10.3)	0.613
Diabetes (%)	2 (7.7)	1 (2.0)	2 (6.9)	0.372
Renal disease (%)	3 (11.5)	2 (3.9)	1 (3.4)	0.417
Autoimmune disease (%)	2 (7.7)	8 (15.7)	2 (6.9)	0.486

ABT (antibiotic), GA (gestational age), intrauterine growth restriction (IUGR), NIV (non-invasive ventilation), IMV (invasive mechanical ventilation), HM (human milk), FHM (fortified human milk), PF (preterm formula), PN (parenteral nutrition), PROM (premature rupture of membranes), * according to pregestational body mass index. Mean ± standard deviation for numerical variables. One-way ANOVA or Kruskal–Wallis test for numerical variables, as appropriate. Fisher’s exact test or Chi-Square test for categorical variables, as appropriate.

**Table 2:** Preterm infant diseases during hospital stay.

Preterm Infant Disease	No ABT (*n* = 26)	3–7 d ABT (*n* = 51)	≥8 d ABT (*n* = 29)	*p*-Value
Culture-confirmed EOS (%)	0	1 (2.0)	1 (3.4)	0.999
Culture-confirmed LOS (%)	0	5 (9.8)	12 (41.4)	<0.0001
Septic shock (%)	0	1 (2.0)	5 (17.2)	0.009
NEC I (%)	3 (11.5)	8 (15.7)	5 (17.2)	0.883
NEC II (%)	1 (3.8)	8 (15.7)	3 (10.3)	0.351
Atypical pneumonia (%)	2 (7.7)	17 (33.3)	12 (42.9)	0.016
Moderate BPD (%)	5 (19.2)	21 (41.2)	17 (58.6)	0.012
Severe BPD (%)	3 (11.5)	8 (15.7)	4 (13.8)	0.937
PDA (%)	6 (23.1)	16 (31.4)	14 (50.0)	0.124
Anemia (%)	10 (38.5)	25 (49.0)	19 (65.5)	0.125
ROP (%)	2 (7.7)	20 (39.2)	16 (55.2)	<0.001
GERD (%)	11 (42.3)	21 (41.2)	10 (34.5)	0.799
Oral intolerance (%)	2 (7.7)	7 (13.7)	5 (17.2)	0.656

ABT (antibiotic), EOS (early-onset sepsis), LOS (late-onset sepsis, NEC (necrotizing enterocolitis), BPD (bronchopulmonary dysplasia), PDA (patent ductus arteriosus), ROP (retinopathy of prematurity), GERD (gastroesophageal reflux disease). Fisher’s exact test or Chi-Square test, as appropriate.

**Table 3:** Neonatal stool pattern from preterm infants <33 weeks GA.

Stool Variable	No ABT (*n* = 26)	3–7 d ABT (*n* = 51)	≥8 d ABT (*n* = 29)	*p*-Value
Meconium start DOL	0.9 ± 0.8	1 ± 1	1 ± 0.8	0.895
Meconium finish DOL	8.5 ± 3.1	9.9 ± 4	13.7 ± 5.1	0.0001
Green stools start DOL	5.5 ± 2.9	6.2 ±2.7	8.2 ± 3.2	0.0027
Yellow stools start DOL	8.2 ± 2.3	9.7 ± 4.1	12.8 ± 5.2	0.0012
Days without BM	1.5 ± 2.34	1.6 ± 1.96	3.2 ± 2.6	0.002

GA (gestational age), ABT (antibiotic), DOL (day of life), BM (bowel movement). Kruskal–Wallis test.

**Table 4:** Multiple linear regression model summary for meconium finish day.

Predictor	B (95% CI)	SE B	Beta	*p*-Value
Model 1 ^a^				
Neonatal ABT	2.597 (1.33, 3.87)	0.639	0.422	0.000
Model 2 ^b^				
Neonatal ABT	2.046 (0.72, 3.38)	0.669	0.332	0.003
Model 3 ^c^				
Neonatal ABT	2.291 (1.00, 3.58)	0.648	0.372	0.001
PROM >18 h	−3.823 (−6.22, −1.42)	1.208	−0.315	0.002
Model 4 ^d^				
Neonatal ABT	2.249 (0.79, 3.71)	0.733	0.365	0.003
PROM >18 h	−3.862 (−6.35, −1.37)	1.252	−0.318	0.003

R^2^ = 0.187 for model 1; ΔR^2^ = 0.058 for model 2; ΔR^2^ = 0.076 for model 3; ΔR^2^ = 0.032 for model 4. ^a^ Model 1: sex (female, male), birth GA classification (extremely preterm, very preterm, moderate preterm), birth weight classification (ELBW, VLBW, LBW), parity (singleton, twins, triplets), and neonatal antibiotic treatment classification (no ABT, 3–7 d ABT, ≥8 d ABT). ^b^ Model 2: same variables as in Model 1 with the addition of IMV, postnatal steroid and surfactant. ^c^ Model 3: same variables as in Model 2 with the addition of chorioamnionitis and PROM >18 h. ^d^ Model 4: same variables as in Model 3 with the addition of enteral feeding start day, neonatal fasting days, neonatal PN days, and feeding regimen classification (HM, HM + FHM, HM + FHM + PF, HM + PF). CI (Confidence Interval), SE (standard error), ABT (antibiotic), GA (gestational age), PROM (premature rupture of membranes), ELBW (extremely low birth weight), VLBW (very low birth weight), LBW (low birth weight), IMV (invasive mechanical ventilation), HM (human milk), FHM (fortified human milk), PF (preterm formula).

**Table 5:** Multiple linear regression model summary for green stools start day.

Predictor	B (95% CI)	SE B	Beta	*p*-Value
Model 1 ^a^				
Neonatal ABT	0.955 (0.13, 1.78)	0.415	0.235	0.024
Birth weight	−1.556 (−2.61, −0.50)	0.531	−0.316	0.004
Model 2 ^b^				
Neonatal ABT	0.855 (−0.04, 1.75)	0.450	0.211	0.061
Birth weight	−1.476 (−2.61, −0.34)	0.573	−0.299	0.012
Model 3 ^c^				
Neonatal ABT	0.921 (0.02, 1.82)	0.453	0.227	0.045
Birth weight	−1–363 (−2.51, −0.22)	0.576	−0.277	0.020
Model 4 ^d^				
Neonatal ABT	0.461 (−0.49, 1.41)	0.476	0.114	0.336
Birth weight	−0.984 (−2.13, 0.16)	0.576	−0.200	0.091
Enteral feeding start day	0.458 (0.001, 0.92)	0.230	0.211	0.050

R^2^ = 0.210 for model 1; ΔR^2^ = 0.005 for model 2; ΔR^2^ = 0.022 for model 3; ΔR^2^ = 0.134 for model 4. ^a^ Model 1: sex (female, male), birth GA classification (extremely preterm, very preterm, moderate preterm), birth weight classification (ELBW, VLBW, LBW), parity (singleton, twins, triplets), and neonatal antibiotic treatment classification (no ABT, 3–7 d ABT, ≥8 d ABT). ^b^ Model 2: same variables as in Model 1 with the addition of IMV, postnatal steroid and surfactant. ^c^ Model 3: same variables as in Model 2 with the addition of chorioamnionitis and PROM >18 h. ^d^ Model 4: same variables as in Model 3 with the addition of enteral feeding start day, neonatal fasting days, neonatal PN days, and feeding regimen classification (HM, HM + FHM, HM + FHM + PF, HM + PF). CI (Confidence Interval), SE (standard error), ABT (antibiotic), GA (gestational age), PROM (premature rupture of membranes), ELBW (extremely low birth weight), VLBW (very low birth weight), LBW (low birth weight), IMV (invasive mechanical ventilation), HM (human milk), FHM (fortified human milk), PF (preterm formula).

**Table 6:** Multiple linear regression model summary for yellow stools start day.

Predictor	B (95% CI)	SE B	Beta	*p*-Value
Model 1 ^a^				
Neonatal ABT	2.461 (1.23, 3.69)	0.619	0.416	0.000
Model 2 ^b^				
Neonatal ABT	2.136 (0.83, 3.44)	0.656	0.361	0.002
Model 3 ^c^				
Neonatal ABT	2.330 (1.03, 3.63)	0.653	0.394	0.001
Model 4 ^d^				
Neonatal ABT	1.754 (0.30, 3.21)	0.732	0.296	0.019

R^2^ = 0.172 for model 1; ΔR^2^ = 0.044 for model 2; ΔR^2^ = 0.039 for model 3; ΔR^2^ = 0.046 for model 4. ^a^ Model 1: sex (female, male), birth GA classification (extremely preterm, very preterm, moderate preterm), birth weight classification (ELBW, VLBW, LBW), parity (singleton, twins, triplets), and neonatal antibiotic treatment classification (no ABT, 3–7 d ABT, ≥8 d ABT). ^b^ Model 2: same variables as in Model 1 with the addition of IMV, postnatal steroid and surfactant. ^c^ Model 3: same variables as in Model 2 with the addition of chorioamnionitis and PROM >18 h. ^d^ Model 4: same variables as in Model 3 with the addition of enteral feeding start day, neonatal fasting days, neonatal PN days, and feeding regimen classification (HM, HM + FHM, HM + FHM + PF, HM + PF). CI (Confidence Interval), SE (standard error), ABT (antibiotic), GA (gestational age), PROM (premature rupture of membranes), ELBW (extremely low birth weight), VLBW (very low birth weight), LBW (low birth weight), IMV (invasive mechanical ventilation), HM (human milk), FHM (fortified human milk), PF (preterm formula).

**Table 7:** Multiple linear regression model summary of DOL to reach 100 mL/kg/day.

Predictor	B (95% CI)	SE B	Beta	*p*-Value
Model 1 ^a^				
Neonatal ABT	3.025 (1.26, 4.79)	0.891	0.348	0.001
Model 2 ^b^				
Neonatal ABT	2.814 (0.89, 4.73)	0.967	0.323	0.005
Model 3 ^c^				
Neonatal ABT	3.098 (1.19, 5.01)	0.960	0.356	0.002
Model 4 ^d^				
Neonatal ABT	1.842 (−0.18, 3.86)	1.017	0.212	0.074
Neonatal PN days	0.187 (−0.02, 0.40)	0.105	0.236	0.077
Neonatal fast days	0.578 (−0.09, 1.24)	0.334	0.208	0.087

R^2^ = 0.209 for model 1; ΔR^2^ = 0.004 for model 2; ΔR^2^ = 0.042 for model 3; ΔR^2^ = 0.122 for model 4. ^a^ Model 1: sex (female, male), birth GA classification (extremely preterm, very preterm, moderate preterm), birth weight classification (ELBW, VLBW, LBW), parity (singleton, twins, triplets), and neonatal antibiotic treatment classification (no ABT, 3–7 d ABT, ≥8 d ABT). ^b^ Model 2: same variables as in Model 1 with the addition of IMV, postnatal steroid and surfactant. ^c^ Model 3: same variables as in Model 2 with the addition of chorioamnionitis and PROM >18 h. ^d^ Model 4: same variables as in Model 3 with the addition of enteral feeding start day, neonatal fasting days, neonatal PN days, and feeding regimen classification (HM, HM + FHM, HM + FHM + PF, HM + PF). CI (Confidence Interval), SE (standard error), ABT (antibiotic), GA (gestational age), PROM (premature rupture of membranes), ELBW (extremely low birth weight), VLBW (very low birth weight), LBW (low birth weight), IMV (invasive mechanical ventilation), HM (human milk), FHM (fortified human milk), PF (preterm formula).

**Table 8:** Multiple linear regression model summary for DOL to reach 150 mL/kg/day.

Predictor	B (95% CI)	SE B	Beta	*p*-Value
Model 1 ^a^				
Neonatal ABT	3.791 (1.77, 5.81)	1.019	0.374	0.000
Birth weight	−3.104 (−5.69, −0.51)	1.304	−0.252	0.019
Model 2 ^b^				
Neonatal ABT	3.726 (1.53, 5.92)	1.104	0.367	0.001
Birth weight	−3.248 (−6.04, −0.46)	1.405	−0.264	0.023
Model 3 ^c^				
Neonatal ABT	4.139 (2.00, 6.27)	1.075	0.408	0.000
Birth weight	−2.834 (−5.55, −0.12)	1.366	−0.230	0.041
Model 4 ^d^				
Neonatal ABT	2.579 (0.30, 4.86)	1.145	0.254	0.027
Birth weight	−1.920 (−4.67, 0.83)	1.385	−0.156	0.169
Neonatal PN days	0.253 (0.02, 0.49)	0.118	0.273	0.035

R^2^ = 0.238 for model 1; ΔR^2^ = 0.006 for model 2; ΔR^2^ = 0.069 for model 3; ΔR^2^ = 0.105 for model 4. ^a^ Model 1: sex (female, male), birth GA classification (extremely preterm, very preterm, moderate preterm), birth weight classification (ELBW, VLBW, LBW), parity (singleton, twins, triplets), and neonatal antibiotic treatment classification (no ABT, 3–7 d ABT, ≥8 d ABT). ^b^ Model 2: same variables as in Model 1 with the addition of IMV, postnatal steroid and surfactant. ^c^ Model 3: same variables as in Model 2 with the addition of chorioamnionitis and PROM >18 h. ^d^ Model 4: same variables as in Model 3 with the addition of enteral feeding start day, neonatal fasting days, neonatal PN days, and feeding regimen classification (HM, HM + FHM, HM + FHM + PF, HM + PF). CI (Confidence Interval), SE (standard error), ABT (antibiotic), GA (gestational age), PROM (premature rupture of membranes), ELBW (extremely low birth weight), VLBW (very low birth weight), LBW (low birth weight), IMV (invasive mechanical ventilation), HM (human milk), FHM (fortified human milk), PF (preterm formula).

**Table 9:** Multiple linear regression model summary for volume of enteral feeds at DOL 14.

Predictor	B (95% CI)	SE B	Beta	*p*-Value
Model 1 ^a^				
Neonatal ABT	−23.194 (−37.61, −8.78)	7.259	−0.333	0.002
Birth weight	23.191 (4.74, 41.64)	9.295	0.274	0.014
Model 2 ^b^				
Neonatal ABT	−20.574 (−36.17, −4.97)	7.854	−0.295	0.010
Birth weight	19.936 (0.07, 39.80)	10.002	0.236	0.049
Model 3 ^c^				
Neonatal ABT	−23.563 (−38.73, −8.40)	7.634	−0.338	0.003
Birth weight	16.939 (−2.34, 36.21)	9.701	0.200	0.084
Model 4 ^d^				
Neonatal ABT	−10.235 (−25.79, 5.32)	7.824	−0.147	0.194
Birth weight	6.992 (−11.82, 25.80)	9.461	0.083	0.462
Neonatal PN days	−2.889 (−4.49, −1.29)	0.805	−0.454	0.001

R^2^ = 0.179 for model 1; ΔR^2^ = 0.009 for model 2; ΔR^2^ = 0.076 for model 3; ΔR^2^ = 0.160 for model 4. ^a^ Model 1: sex (female, male), birth GA classification (extremely preterm, very preterm, moderate preterm), birth weight classification (ELBW, VLBW, LBW), parity (singleton, twins, triplets), and neonatal antibiotic treatment classification (no ABT, 3–7 d ABT, ≥8 d ABT). ^b^ Model 2: same variables as in Model 1 with the addition of IMV, postnatal steroid and surfactant. ^c^ Model 3: same variables as in Model 2 with the addition of chorioamnionitis and PROM >18 h. ^d^ Model 4: same variables as in Model 3 with the addition of enteral feeding start day, neonatal fasting days, neonatal PN days, and feeding regimen classification (HM, HM + FHM, HM + FHM + PF, HM + PF). CI (Confidence Interval), SE (standard error), ABT (antibiotic), GA (gestational age), PROM (premature rupture of membranes), ELBW (extremely low birth weight), VLBW (very low birth weight), LBW (low birth weight), IMV (invasive mechanical ventilation), HM (human milk), FHM (fortified human milk), PF (preterm formula).

**Table 10:** Multiple linear regression model summary for volume of enteral feeds at DOL 28.

Predictor	B (95% CI)	SE B	Beta	*p*-Value
Model 1 ^a^				
Neonatal ABT	−16.292 (−32.71, 0.13)	8.271	−0.212	0.052
Sex	29.156 (6.87, 51.44)	11.224	0.256	0.011
Model 2 ^b^				
Neonatal ABT	−14.014 (−31.38, 3.36)	8.746	−0.182	0.113
Sex	29.664 (6.99, 52.34)	11.415	0.261	0.011
IMV	−29.271 (−57.79, −0.75)	14.359	−0.257	0.044
Surfactant	17.066 (−8.37, 42.50)	12.806	0.149	0.186
Model 3 ^c^				
Neonatal ABT	−17.020 (−33.94, −0.10)	8.518	−0.221	0.049
Sex	26.708 (4.53, 48.89)	11.163	0.235	0.019
IMV	−30.353 (−58.50, −2.20)	14.170	−0.267	0.035
Surfactant	26.031 (0.75, 51.32)	12.728	0.228	0.044
Model 4 ^d^				
Neonatal ABT	6.395 (−8.87, 21.66)	7.680	0.083	0.407
Sex	14.822 (−4.09, 33.74)	9.514	0.130	0.123
IMV	−26.793 (−50.27, −3.31)	11.811	−0.236	0.026
Surfactant	21.865 (0.76, 42.97)	10.616	0.191	0.042
Enteral feeding start day	7.769 (0.39, 15.15)	3.712	0.189	0.039
Neonatal PN days	−2.098 (−3.67, −0.53)	0.790	−0.299	0.009
Neonatal fast days	−12.010 (−17.03, −6.99)	2.525	−0.489	0.000

R^2^ = 0.126 for model 1; ΔR^2^ = 0.048 for model 2; ΔR^2^ = 0.074 for model 3; ΔR^2^ = 0.297 for model 4. ^a^ Model 1: sex (female, male), birth GA classification (extremely preterm, very preterm, moderate preterm), birth weight classification (ELBW, VLBW, LBW), parity (singleton, twins, triplets), and neonatal antibiotic treatment classification (no ABT, 3–7 d ABT, ≥8 d ABT). ^b^ Model 2: same variables as in Model 1 with the addition of IMV, postnatal steroid and surfactant. ^c^ Model 3: same variables as in Model 2 with the addition of chorioamnionitis and PROM >18 h. ^d^ Model 4: same variables as in Model 3 with the addition of enteral feeding start day, neonatal fasting days, neonatal PN days, and feeding regimen classification (HM, HM + FHM, HM + FHM + PF, HM + PF). CI (Confidence Interval), SE (standard error), ABT (antibiotic), GA (gestational age), PROM (premature rupture of membranes), ELBW (extremely low birth weight), VLBW (very low birth weight), LBW (low birth weight), IMV (invasive mechanical ventilation), HM (human milk), FHM (fortified human milk), PF (preterm formula).

## Data Availability

The data presented in this study are available on request from the corresponding author.
